# Functional Role of circRNAs in the Regulation of Fetal Development, Muscle Development, and Lactation in Livestock

**DOI:** 10.1155/2021/5383210

**Published:** 2021-02-19

**Authors:** Tianle He, Qingyun Chen, Ke Tian, Yinzhao Xia, Guozhong Dong, Zhenguo Yang

**Affiliations:** ^1^Laboratory for Bio-Feed and Molecular Nutrition, College of Animal Science and Technology, Southwest University, Chongqing, China; ^2^The United Graduate School of Agricultural Science, Gifu University, Gifu, Japan

## Abstract

circRNAs are a class of endogenous noncoding RNA molecules with closed loop structures. They are mainly responsible for regulating gene expression in eukaryotic cells. With the emergence of high-throughput RNA sequencing (RNA-Seq) and new types of bioinformatics tools, thousands of circRNAs have been discovered, making circRNA one of the research hotspots. Recent studies have shown that circRNAs play an important regulatory role in the growth, reproduction, and formation of livestock products. They can not only regulate mammalian fetal growth and development but also have important regulatory effects on livestock muscle development and lactation. In this review, we briefly introduce the putative biogenic pathways and regulatory functions of circRNA and highlight our understanding of circRNA and its latest advances in fetal development, muscle development, and lactation biogenesis as well as expression in livestock. This review will provide a theoretical basis for the research and development of related industries.

## 1. Introduction

Circular RNAs (circulRNAs, circRNAs) are widely found in eukaryotic cells; they are a special type of nucleotide sequences containing conserved microRNAs (miRNAs) binding sites [1]. Studies have shown that most of the circRNAs currently found are noncoding RNAs that do not encode proteins, but they play as a function at specific stages of biological tissue differentiation and development [2]. In order to explore the effects of circRNAs on fetal development, muscle development, and milk formation quality in livestock, scholars have done a lot of basic research.

On the one hand, some studies have shown that circRNAs play an important role in regulating the lactation of livestock [3–5]. Some studies detected a total of 37,818 circRNAs in goat breast tissue, of which 864 circRNAs were differentially expressed, 611 were upregulated, and 253 were down-regulated [6], so they speculated that these circRNAs may be related to breast development and lactation. Furthermore, studies identified 4,048 circRNAs in the mammary glands of cows on the day 90 and 250 postpartum by RNA sequencing (RNA-seq), of which only 2,231 circRNAs were expressed simultaneously in both day 90 and day 250 [7], suggesting that circRNAs may have a higher stage specificity during lactation. In addition, among the 4,906 circRNAs detected in mammary gland (MG) tissues of Small-Tailed Han (STH) sheep and Gansu Alpine Merino (GAM) sheep parenchyma at peak lactation, 33 circRNAs were expressed differentially, including 18 upregulated circRNAs and 15 downregulated circRNAs [8]. Five circRNAs (circ-19403, circ-015111, circ-014121, circ-007220, and circ-004632) were only expressed in the MG of the STH sheep and 4 circRNAs (circ-004110, circ-021253, circ-017116, and circ-003297) were only expressed in the MG of the GAM sheep [8].

On the other hand, a large number of studies have shown that circRNAs play an important role in regulating muscle development of livestock [2, 9–11]. Sun et al. found that 4,360 candidate circRNA were differentially expressed in the longissimus dorsi of Landrace and Lancang pigs. Of these, 1,401 circRNAs were upregulated in the Lantang library, while 2,959 circRNAs were downregulated. Of the differentially expressed circRNAs, 236 candidates were edited from 93 functional hosting genes related to myogenesis, inspiring us that circRNAs can play a regulatory role in animal muscle development [12]. Wang et al. showed that circTTN can activate IGF-2/phosphatidylinositol 3 kinase (PI3K)/AKT signaling pathway through competitive binding with miR-432 and promote the proliferation and differentiation of bovine primary myoblasts, and their further studies have shown that overexpression and inhibition of circTTN can jointly induce its role in promoting the proliferation and differentiation of bovine primary myoblasts [13].

In addition, studies have shown that circRNAs can regulate fetal development, and their expression is the most abundant and complex in the cerebral cortex on the 60th day of pregnancy [14–16]. Studies have shown that there are 10,032 circRNAs in human ocytes and embryos, a small portion of which are maternally expressed, and most of the circRNAs have been shown to be related to animal embryo development [17]. Our previous studies have shown that high-energy diets jointly regulate placental nutrient transport and fetal development in Yorkshire gilts through feedback between circ-Amotl1 and miR-17-5p [18]. It can be seen that circRNAs have a specific effect on fetal development. Here, we will focus on the important molecular mechanism and mode of action of circRNAs in the development of animal husbandry. The purpose of this study is to provide researchers with more circRNA-related theories to guide the development of animal husbandry.

## 2. Overview of circRNAs

### 2.1. Discovery and Classification of circRNAs

circRNAs were first extracted from several RNA viruses in the 1970s [19], but for a long time, they were considered to be mRNA transcription errors because of their low abundance in organisms. Subsequently, circRNAs were successively found to exist in archaea, nematodes, zebrafish, mice, and human cells and considered to do not encode proteins [19]. With the development of science and technology as well as the advent of new computational methods for nonpolyadenylated RNA transcription, circRNAs have begun to be highly valued, and circRNAs have gradually become one of the research hotspots. Recent studies have shown that circRNAs play an important role in various physiological and pathological processes and can even serve as templates for protein translation [20]. In order to carry out follow-up research in a more organized way, circRNAs could be categorized into three types, including exon circRNAs, intron circRNAs, and exon and intron mixed circRNAs based on the genomic loci and the relationship with the connected parental transcript [21–23].

### 2.2. The Formation of circRNAs

**Figure 1:**
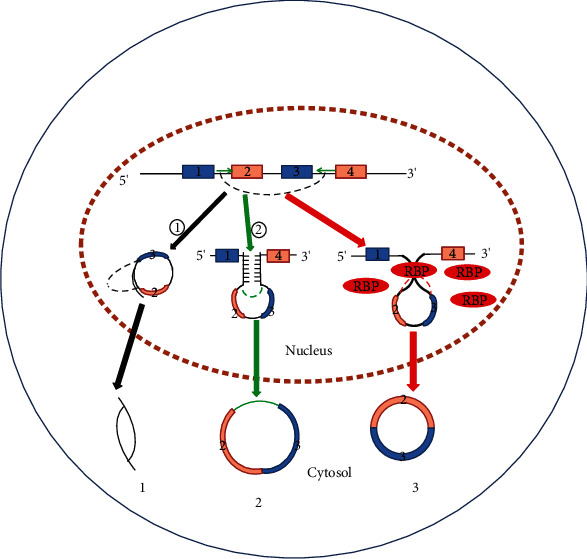
The main formation of circRNAs: 1: circRNAs are formed after introns in the lasso structure are excised; 2: intron pairing driving cyclization, thus forming circRNAs; 3: the RNA-binding protein specifically binds to flanking introns at both ends of the RNA, thus forming circRNAs.

Recent studies have shown that circRNAs are mainly formed by reverse splicing of RNA into loops [24], and most eukaryotic circRNAs are formed by classical spliceosome or group I/II ribozymes [24–26]. Li et al. pointed out two hypotheses for the formation of circRNAs: first, the lasso structure drives cyclization, and the lasso structure is a by-product of exon hopping. After the intron in the lasso structure is removed, exons can be connected to form circRNAs; the second is intron pairing driving cyclization, and there is an intron with reverse complementary sequence at both ends of the ring-shaped exon. The pairing mediation of the reverse complementary sequence of the intron can make the splicing donor and splice recipient of the exon spliced into a ring closer to each other in space, thus forming circRNAs [21]. Pagliarini et al. found that RNA-binding proteins can also specifically bind to flanking introns at both ends of RNA, which can reduce the distance between splice and donor acceptor, finally form circRNAs [27, 28] (see Figure 1). In addition, CDR1as can also be generated by mammalian-wide interspersed repeats (MIRs) in mammals [29]. These findings suggest that the formation of circRNAs may be affected by many other factors. Therefore, more research is needed to fully understand the formation of circRNAs.

### 2.3. Characteristics of circRNAs and Their Biological Functions

According to the research results in recent years, the characteristics of circRNAs can be summarized as follows:

1. Localization specificity: most circRNAs are exonic circular RNAs (EcircRNA), which are mainly present in the cytoplasm, and only a few circular intronic RNAs (CiRNA) and exon-intron circular RNAs (EIciRNA) containing introns exist in the nucleus of eukaryotic cells [30].
2. Expression specificity: medical studies have shown that circRNAs have certain tissue and disease specificity [30].
3. Coding specificity: noncoding RNA can only play a regulatory role during transcription or post-transcription without coding protein formation [1], but a few can encode proteins [31, 32].
4. Structural stability: circRNAs do not have 5′ and caps and 3′ end polyA tails and are not easily degraded by exonuclease RNAseR [33, 34].
5. circRNAs: they are highly conserved in many species, such as humans, mice, nematodes, and zebrafish [21], and only a small number of them can rapidly evolve and change [34].
6. Abundance and diversity: only circRNAs found in eukaryotic cells have exceeded 20,000 species [1], a few are formed by direct cyclization of introns, and most of them come from exons [35].

Confirmed or speculated through research, circRNAs control gene expression at different levels, including transcription, pre-mRNA splicing, mRNA translation, and protein function. In recent years, more researches have focused on that circRNAs can act as sponges for miRNAs. When circRNAs specifically bind to multiple miRNAs, they can release the inhibitory effect of miRNAs on their targeted mRNAs, thereby promoting the transcription and protein translation of targeted genes [36–41]. It has also been reported that circRNAs can be spliced with linear RNA, thereby smoothly “capturing” mRNA and then regulating the life process [42]; circRNAs can also interact with RNA polymerase II (Pol II), thereby promoting the transcription of parent genes [31]. Some circRNAs can regulate the normal physiological activities of biological individuals by participating in the synthesis of body proteins [43, 44]. For example, circ-Amotl1 physically binds to both 3-phosphoinositide-dependent protein kinase 1 (PDK1) and protein kinase B (AKT), facilitating the cardioprotective nuclear translocation of pAKT [45]. Very few circRNAs can be used as translation templates to promote translation [44]. Recently, some studies have shown that circRNAs can also be used as biomarker molecules [46, 47] and are currently used as markers for the diagnosis of related diseases [45].

### 2.4. circRNAs Regulate Fetal Development in Livestock

Mammalian fetal development refers to the process by which fertilized eggs develop into larvae, and the fetus is regulated by many factors during early development. Under the joint control of multiple factors, fertilized eggs can successfully divide and differentiate into normal fetuses. In the production of animal husbandry, healthy livestock fetuses are the foundation of population continuity and industrial development. The higher the number and health of livestock fetuses, the greater the value of breeding and economic value. Therefore, research on regulating fetal development in mammals is essential. Some scholars have found through deep RNA sequencing and bioinformatics analysis that the expression of circRNAs has tissue-cell specificity and stage-specific developmental differentiation during the embryonic stage of animals [48, 49]. Many recent studies have shown that circRNAs may have unique and important functions during embryonic development, and related studies on the long-term development of the fetus after birth are gradually increasing with the placenta-based microenvironment of the fetal interface [49]. The researchers found that circRNAs play an important role in a variety of biological processes, including cell differentiation and development, and mammalian pregnancy is accompanied by cell differentiation and development of individual fetuses, which indicates that circRNAs are very likely to growth and development [19, 50]. Dang et al. studied human embryonic development and found that there were 1,032 types of circRNAs in the exon region of 2,974 genes in cells (endocell clusters, nourishing ectoderm cells, etc.) at different developmental stages [15]. Most circRNAs can perform specific dynamic regulation during embryonic development and are mainly involved in the regulation of material metabolic pathways in early embryonic development [15]. The development of a mammalian fetal brain is a crucial step for embryonic development. Szabo et al. have pointed out in the study of human fetal brain development that circRNAs in some areas of the fetal brain (such as the frontal cortex) are significantly enriched and these circRNAs are important for studying human embryonic development [48]. Wang et al. selected 5 brain tissues from 6 time points of fetal pig development to detect the expression of circRNAs [14]. They found that the sow's quantity and complexity of circRNAs expression were most obvious in the cortex at the 60th day of pregnancy of the 13,854 expressed genes detected; 2,195 genes expressed 4,634 unique circRNAs; In addition, Hirotsu et al. found that the expression of circRIMS2 in the embryonic pig cortex reached a peak during development, suggesting that circRNAs are one of the major factors regulating mammalian embryonic brain development [51]. Shen et al. used adult intrauterine growth restriction (IUGR) pigs and normal pigs as models and used RNA-Seq technology to detect the expression of circRNAs in the liver during pregnancy [52]. A total of 403 circRNAs were found, of which 44 had differences in expression (*p* < 0.05); their study showed people a variety of circRNAs that may be involved in the development of IUGR pigs, laying a foundation for future studies of fetal development during pregnancy [52]. Fan et al. sequenced 913 new linear transcripts and 2,891 circRNAs found in mouse preimplantation embryos by SUPeR-seq, analyzed the abundance of circRNAs, the function of enriched genes, and the characteristics of circRNAs sequence during development, and speculated that these circRNAs may play a role in the embryonic development of other animals [53]. Zhang et al. constructed circRNA miRNA during the implantation period of dairy goat embryos and found that circRNA8073 (cir8073) can reduce the level of miR-181a by acting as a miRNA sponge [54]. This effect indirectly increases the expression of neurotensin in endometrial epithelial cells (EECS), thereby inhibiting the apoptosis of EECS during embryo implantation, which is conducive to embryo implantation and embryo development. In addition, Xu et al. speculated that the tissue-specific and developmental specificity of circRNAs in adults and fetuses may be determined by different expression levels of circRNAs in tissues after a large number of studies, but there has been no new breakthrough in this area [55]. Other studies have reported that circRNAs also play important roles in gametogenesis and preeclampsia [56, 57]. Studies in pigs have also shown that circRNAs are expressed at different levels in fetuses with different body weights and attachment sites [16]. Researchers showed that many miRNAs in animals can be preferentially expressed in the placenta and are related to pregnancy and birth circRNAs are directly related to the regulation of miRNAs expression [58]. Therefore, the researchers speculated that circRNAs and miRNAs were affected during the placental nutrient transport comprehensive regulatory role. In fact, as early as 2013, Hansen found that miRNAs play important regulatory functions in animal embryonic development [36]. Maternal high energy diet during pregnancy could promote placental nutrient transport and accelerate fetal intrauterine growth in the third trimester of pregnancy [18]; the study of sows finally confirmed our conjecture; we measured the expression of circ-Amotl1 and miR-17-5p in the placenta of sows on the 90th day of pregnancy. The results showed that the expression level of circ-Amotl1 in placenta at the end of uterine horn in the low energy group and high energy group was significantly higher than that in the cervix, while the expression level of miR-17-5p was significantly decreased in the low energy group and high energy group [18]. Finally, we found that the uniform distribution of nutrition and fetal development at different placental attachment sites were affected by circ-Amotl1 and miR-17-5p; it is suggested that the interaction between circ-Amotl1 and miR-17-5p can regulate the ability of maternal placental nutrient transport and then regulate embryonic development [18]. The results of this series of studies have inspired us that circRNAs are likely to have an important effect on embryonic development through the sponge effect of miRNAs. The regulation of circRNAs on mammalian embryonic development is also becoming more and more obvious, but there are few studies on domestic animals. Therefore, scientific researchers need to make persistent efforts based on previous research.

### 2.5. Regulation of circRNAs on Muscle Development in Livestock

**Figure 2:**
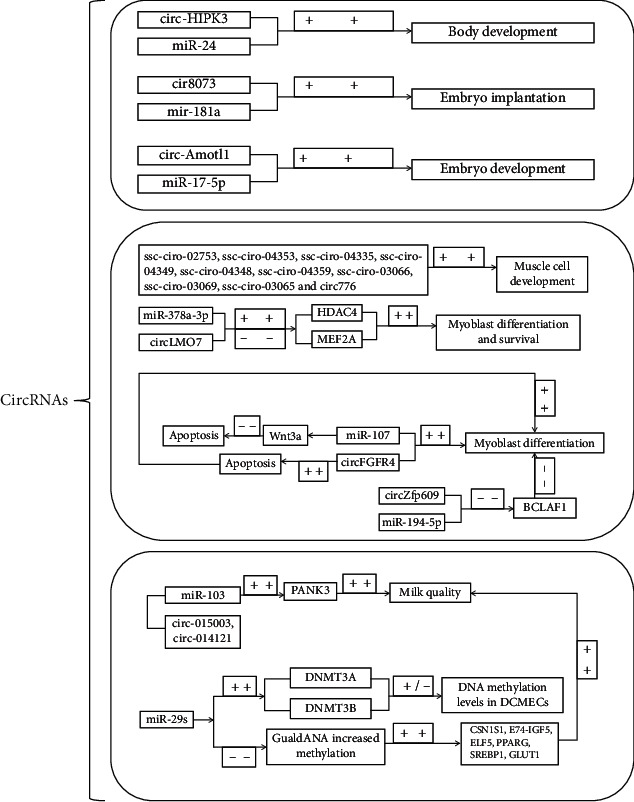
“+ +” means promotion, “- -” means inhibition, and the arrow points to the regulation direction.

Animal meat is rich in protein, with a complete range of essential amino acids, and the proportion of each amino acid is appropriate, which is close to the protein composition of the human body. It has been reported that circRNAs can play a pivotal role in muscle development through specific miRNAs. Researchers have found that the number of circRNAs in animal skeletal muscle and myoblasts ranges from 2,000 to 37,000 [59–65]. Cao et al. identified circRNAs of the longest muscle in sheep and found that many circRNAs such as circ776 can interact with specific miRNAs for muscle growth and development [66]. After further research, they found that the host genes of circRNAs are involved in muscle cell development and related signaling pathways. Liang et al. comprehensively analyzed circRNAs in three skeletal muscles of Guizhou mini pigs (S. scrofa), identified 149 circRNAs that may be related to muscle growth, and found that their host genes were significantly involved in muscle development, contraction, chromatin modification, cationic homeostasis, and ATP hydrolysis coupled proton transport [62]. After deep research, they found ssc-ciR-02753, ssc-ciR-04353, ssc-ciR-04335, ssc-ciR-04349, ssc-ciR-04348, ssc-ciR-04359, ssc-ciR-03066, ssc-ciR-03069, and ssc-ciR-03065 can regulate muscle growth by affecting cell proliferation and fusion during early postpartum muscle development [62]. The researchers also pointed out that circLMO7 can bind to miR-378a-3p to cause HDAC4 to increase and MEF2A expression to decrease and ultimately promote bovine myoblast differentiation and survival [63]. Peng et al. found that the expression level of circSNX29 in bovine embryonic skeletal muscle was significantly higher than that of adult skeletal muscle [67]. The overexpression of circSNX29 can inhibit myoblast proliferation and promote myoblast differentiation. Further studies have shown that circSNX29 can bind to its target miR-744 and keep miR-744 away from Wnt5a, resulting in increasing mRNA expression level of Wnt5a and phosphorylation level of protein kinase C(PKC), thus effectively activating Wnt5a/Ca^2+^ signal pathway and regulating embryonic bovine muscle development. Other studies have shown that circFUT10 produced by FUT10 is mainly expressed in bovine skeletal muscle tissue. When circFUT10 is overexpressed, it can inhibit cell proliferation, induce myoblast apoptosis, and ultimately promote myoblast differentiation [68]. Li et al. pointed out that circFGFR4 is highly expressed in bovine skeletal muscle and overexpression of circFGFR4 can also cause apoptosis, thereby promoting myoblast differentiation [69]. RNAhybrid and TargetScan showed that circFGFR4 contains 18 putative miR-107 binding sites, and luciferase experiments and RNA pull-down confirmed the interaction between miR-107 and circFGFR4, and they found that Wnt3a was miR-107 target, while Wnt3a can inhibit myotube formation and protect myoblasts from apoptosis. However, whether this is a major feature of Wnt3a remains to be verified. Based on the above studies, Siengdee et al. pointed out that miR-194 can be upregulated during porcine C2C12 myoblast differentiation [70]. Although studies have shown that miR-194-5p and circZfp609 (circRNA) have four binding sites, when circZfp609 binds to miR-194-5p, it can inhibit BCLAF1 (Bcl2-associated transcription factor 1) [71], which can inhibit further differentiation of muscle cells. However, there are no reports on whether the combination of circZfp609 and miR-194-5p can affect the differentiation of livestock myoblasts. Thus, it can be seen that circRNAs can regulate the muscle development of livestock through CeRNA (circRNA-miRNA-mRNA) coexpression network (see Figure 2).

Many studies have shown that animal muscle formation is regulated by RNA-binding proteins and long-chain noncoding RNA (ncRNA) at the posttranscriptional level [72, 73]. Circ-ZNF609 can initiate translation and directly participate in muscle cell development through a mechanism independent of cap structure [74]. Pandey et al. [75] indicated that binding of Pur protein to CircSAMD4 can promote muscle development by reducing MHC transcription. Pura and Purb are myogenic inhibitors that inhibit transcription of the myosin heavy chain (MHC) protein family. Silencing of circsamD4 enhances the binding of the PUR protein to the MHC promoter, while overexpression of circsamD4 interferes with the binding of the PUR protein to the MHC promoter, suggesting that circsamD4 can bind to the PUR protein and prevent its interaction with DNA. These effects were cancelled when the mutant circSamd4 without the PUR binding site was used. Yin et al. [76] found that circFAM188B contains an open reading frame (ORF), which can be directly translated into circFAM188B-103aa and thus directly promote the development of skeletal muscle in broilers. Thus, it can be seen that circRNAs can also regulate the muscle development of livestock and poultry at the transcriptional level.

circRNAs are one of the more comprehensive studies of ncRNAs, which are particularly outstanding in regulating animal muscle development. The above series of research results inspired us that circRNAs are involved in the regulation of muscle development. Although some of the research on the regulation mechanism is not clear, in general, most of the current studies on circRNAs to regulate muscle formation have turned to the research of related miRNAs. We find that many researchers are trying to explain the related physiological phenomena by studying the interaction between circRNAs and miRNAs, but the specific binding of circRNAs and miRNAs is only a small part of the regulation of life activities by circRNAs (see Figure 2).

### 2.6. Regulation of circRNAs on Lactation and Milk Quality in Livestock

In recent years, a large number of studies have shown that circRNAs play an important role in regulating the lactation of livestock [5, 8, 77, 78]. Lin et al. found that the expression of miR-103 was related to lactation through high-throughput sequencing [79]. Further functional analysis showed that the overexpression of miR-103 in mammary epithelial cells increased the transcription of genes related to milk fat synthesis, resulting in the formation of fat droplets, the accumulation of triglycerides, and the upregulation of the proportion of unsaturated fatty acids [79]. In addition, studies have shown that miR-103 is corelated with one upregulated circRNA (circRNA_007873) and two downregulated circRNAs (circRNA_010763, cir-cRNA_015622) at the same time [80]. However, there is still a lack of direct evidence that circRNAs and miR-103 work together to regulate goat lactation. Zhang et al. found that circRNAs are abundant in the mammary glands of cows, and the expression of circRNAs in the mammary glands of cows is different at different lactation stages [7]; they reported that the content and specificity of circRNAs in bovine mammary glands at 90 and 250 days postpartum were different and specific. The 90-day and 250-day periods corresponded to the prelactation period and the postlactation period, respectively. The comparison showed that various nutrient contents of dairy cows changed significantly during the two lactation periods. It is well known that casein is an important indicator of milk quality assessment, also a major protein in mammals including cows, sheep, and human milk, and an important source of amino acids and calcium and phosphorus for young children [81]. Zhang et al. found four casein-binding genes in milk, namely, *α*s1-casein gene (CSN1S1), *α*s2-casein gene (CSN1S2), casein *β* gene (CSN2), and casein k gene (CSN3), can produce circRNAs in bovine mammary glands, and the abundance of circRNAs expressed by CSN1S1 and CSN2 on the 90th day of lactation was significantly higher than that on the 250th day of lactation. Interestingly, the abundance of circRNAs is consistent with the change in the proportion of casein in milk during lactation [7]. The above study inspired us that circRNAs have an important relationship with milk production and milk quality in dairy cows. Combining the discovery that the miR-2284 family is expressed at high levels in bovine mammary epithelial cells [82], Zhang et al. pointed out that bovine casein circRNAs have high-density binding sites for the miR-2284 family and they also predicted miR-2284 targets; these are mRNAs of CSN1S1 and CSN2, and casein circRNAs may act as miR-2284 sponges to regulate casein translation [7]. In addition, Ma et al. sequenced lactating (early and mature) goat breast tissues and found that circRNAs can form ceRNAs (circRNA-miRNA-mRNA) together with mRNA and miRNAs to participate in the regulation of goat lactation [6]. That is to say, circRNA establishes a functioning system related to mRNA through the intermediate targeting of miRNA, thereby forming a network system in which circRNAs regulate the translation process of mRNA. Therefore, the ceRNAs (circRNA-miRNA-mRNA) established by Ma et al. based on previous studies provide a new research idea for analyzing the specific regulation of a gene in related biological processes [6]. This idea currently has an irreplaceable role in studying circRNAs as miRNA sponges. Bian et al. found that miR-29s regulates DNA methylation levels in DCMECs by reverse targeting DNMT3A and DNMT3B, and the inhibitory effect of miR-29 will cause GualdANA hypermethylation, increase methylation level, and promote important lactation related genes (casein phosphatase 1 (CSN1S1), E74-IGF5 (ELF5), peroxisome proliferator-activated receptor gamma (PPARG), sterol regulatory element binding protein-1(SREBP1), and glucose transporter 1 (GLUT1)) and further regulate the secretion of milk proteins, triglycerides, and lactose in dairy mammary epithelial cells [83]. Wright et al. also found that many miRNAs are involved in the regulation of milk protein synthesis and mammary gland development in dairy cows [84]. Through the above studies, we found that circRNAs play an important role in regulating milk production and milk quality in livestock. Combining with the ceRNA system established by Ma and others [6], we can speculate that the lactation process of cows similar to those regulated by mir-29s will be affected by corresponding circRNAs, and Hao et al. analyzed the constructed miRNA-circRNA network and found that 43 miRNAs related to mammary gland development and lactation have a targeting relationship with circ-001091 [8]. Network analysis also shows that circ-014121 and circ001091 have miR-29a and miR-29b targets, and this finding just confirms our conjecture.

To date, little research has been done on circRNAs in breast tissue. Especially in related species, the expression profile of circRNAs has only been reported in cows and goats. However, the above research results are sufficient to show that circRNAs have important regulatory effects on animal milk production, milk protein synthesis, and mammary gland development. This lays a foundation for us to explore the regulation of milk formation and milk quality by circRNAs. However, the related pathways of circRNAs to regulate milk formation and milk quality are not very clear, so exploring this series of laws has become one of the issues that need to be solved in the development of animal husbandry.

## 3. Conclusions

The discovery of circRNAs has greatly broadened our understanding of gene expression regulatory mechanisms. With the development of science and technology and the unremitting efforts of researchers, we will know more and more about circRNAs. As a small molecular substance with biological regulatory functions widely exists in organisms, circRNAs will become a hot area in RNA regulatory networks. In recent years, more and more evidence shows that circRNAs can regulate fetal development, muscle development, and lactation of livestock to some extent. Through the study of circRNAs, researchers can reunderstand and “manipulate” livestock fetal development, muscle development, and lactation at the genetic level, which can help improve livestock production and reproduction performance. However, at present, we are not very clear about the regulatory mechanism of circRNAs, and even some of these regulatory pathways remain only at the guessing stage. Therefore, researchers need to strengthen the research in this area to enrich the regulation theory of animal product formation and growth and development and lay the foundation for the development of agricultural economy.

## Data Availability

Not applicable.
